# Supplementary material for: Breaking constraint of mammalian axial formulae
Source: Nat Commun. 2022 Jan 11;13:243. doi: 10.1038/s41467-021-27335-z (PMC8752674; doi:10.1038/s41467-021-27335-z)
Supplement: Supplementary file 1 — Supplementary Information [file 41467_2021_27335_MOESM1_ESM.pdf]

List of Supplementary Information

Supplementary Text

- Supplementary note on Hox13 mouse mutant phenotype
- Link to Supplementary Movies

Supplementary Figures 1-6

Supplementary Tables 1-6

Supplementary Text

Supplementary note on Hox13 mouse mutant phenotype

The work of Economides and colleagues, genetically deleting *Hoxb13* in the mouse[16], demonstrated an overgrowth phenotype of the caudal spinal cord and caudal/tail vertebrae. Based on image 2B in this publication, subsequent publications have cited this phenotype as additional tail elements, not solely larger elements. However, even on isogenic mouse backgrounds, we and others have demonstrated variation in tail vertebrae number +/-1 around a mean, thus it is critical to perform appropriate quantification to validate any increase in total vertebral number. Quantification was not performed in Economides et al., and thus we have not cited this work as a known mechanism that increases vertebral number. Similarly, analysis of *Hoxc13* mouse mutants was not able to quantify an increase in tail vertebral number over the variation seen between individuals[17], and further analysis of both mouse mutants is required before conclusions are drawn.

Link to Supplementary Movies

https://figshare.com/s/78383c7beb9b85c959bf

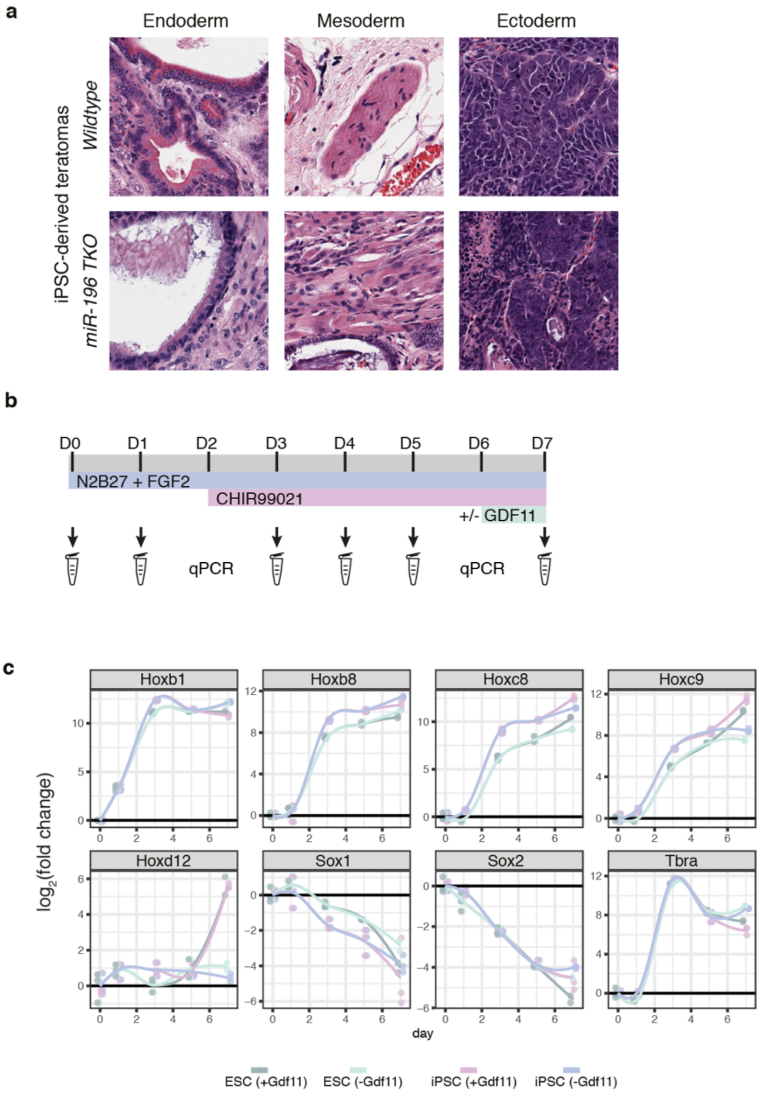

Supplementary Figure 1: Characterisation of pluripotent stem cell tools before and during *in vitro* differentiation to axial progenitors.

a, *In vivo* teratoma assay of WT and miR-196-triple Knockout (TKO) induced pluripotent stem cell (iPSC) lines generated for these studies confirms both cell lines are competent to produce derivatives of all three germ layers. **b**, Schematic of 7-day (D= day) *in vitro* differentiation protocol employed. **c**, Comparison of axial identity and cell lineage marker gene expression between WT embryonic stem cell (ESC) and WT iPSC lines used in these studies. Quantitative PCR (qPCR) analysis of select genes including anterior (*Hoxb1*), trunk (*Hoxb8*, *Hoxc8*) and posterior (*Hoxd12*) *Hox* genes, NMP/early mesoderm marker *T-Brachyury* (*T-Bra*) and the neural markers *Sox1 and Sox2* showed near-identical expression kinetics for all genes assessed over the course of differentiation. Plotted is the log_2(fold change) relative to Day 0, 3 biological replicates for each condition and day, fitted with a curve using local polynomial regression fitting. Source data are provided as a Source Data file.

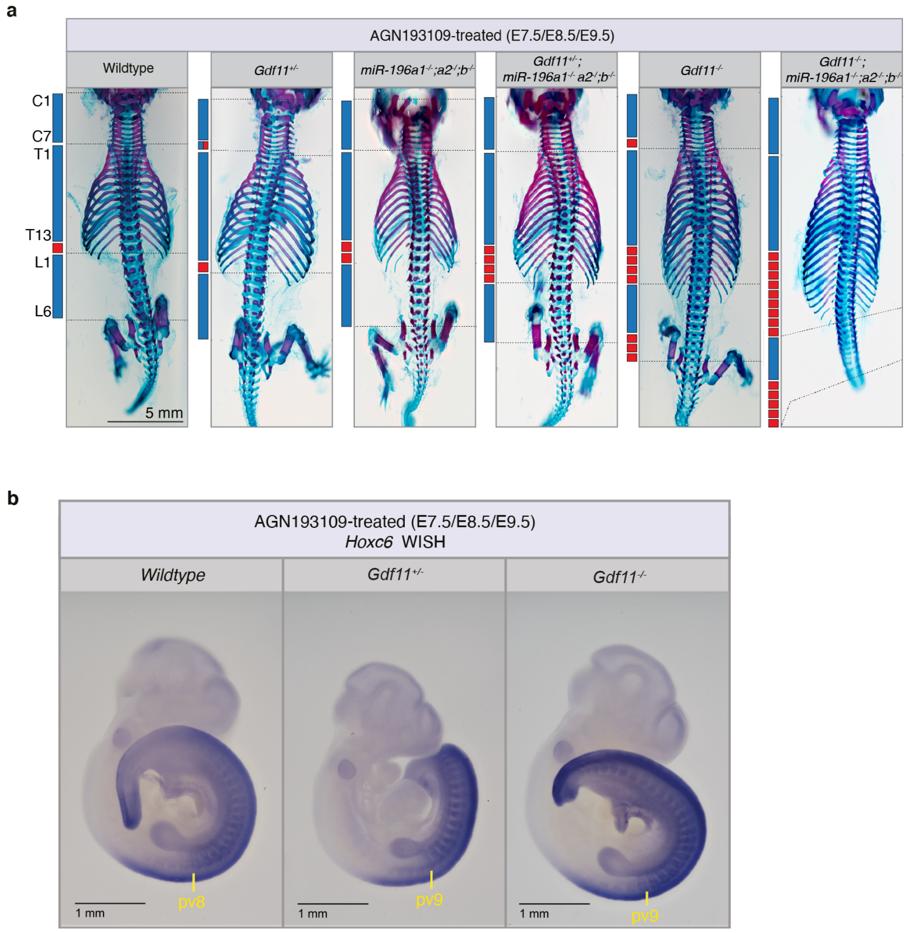

Supplementary Figure 2: Inhibition of retinoic acid (RA) receptors in utero further changes axial formulae and together with Gdf11 controls the anterior expression boundary of Hoxc6.

a, Representative Embryonic day (E)18.5 skeletal preparations across *miR-196* and *Gdf11* individual and compound mouse mutant skeletal phenotypes focusing on the presacral vertebral column following AGN193109 treatment. C= cervical; T= thoracic; L= lumbar. **b**, Whole mount *in situ* hybridisation (WISH) analysis of *Hoxc6* expression in WT, *Gdf11*$^{+/-}$ and *Gdf11*$^{-/-}$ E10.5 embryos exposed to AGN193109 in utero. The combined decrease in Gdf11 and RA signalling led to a posterior shift in the rostral boundary of *Hoxc6* expression by one somite. pv= prevertebra.

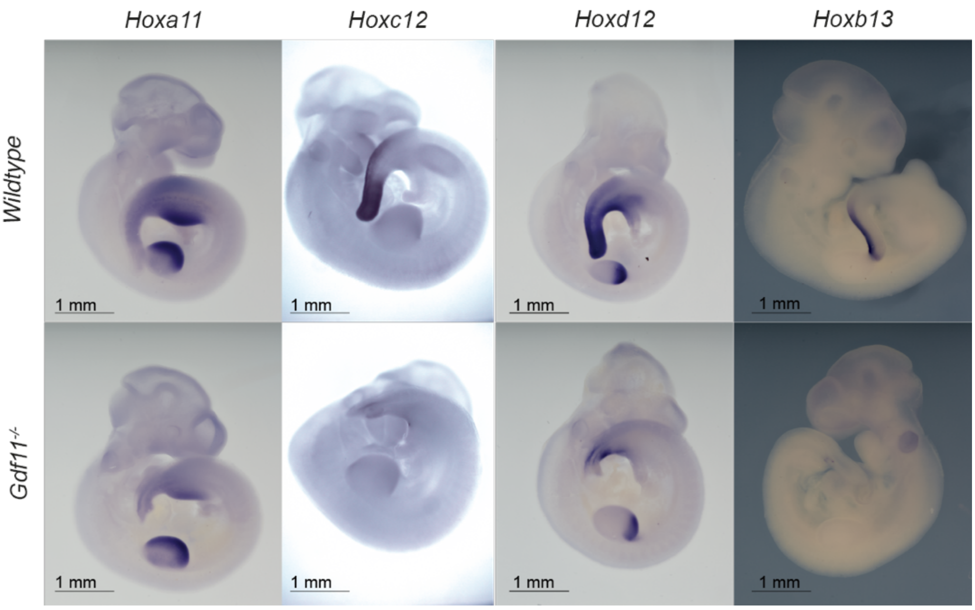

Supplementary Figure 3: Validation of altered *Hox* gene expression identified using Fluidigm, with spatial context provided by *in situ* hybridisation.
Spatial characterisation of selected posterior *Hox* genes by whole mount *in situ* hybridisation of E10.5 WT and *Gdf11*$^{-/-}$ embryos confirms quantitative changes identified by Fluidigm PCR .
Hoxa11 expression is cleared from the tail bud in WT at this stage of development, however expression extends to the tail tip in *Gdf11*$^{-/-}$ embryos. All other posterior Hox genes assessed showed a significant reduction or complete lack of expression in *Gdf11*$^{-/-}$ embryos when compared to WT.

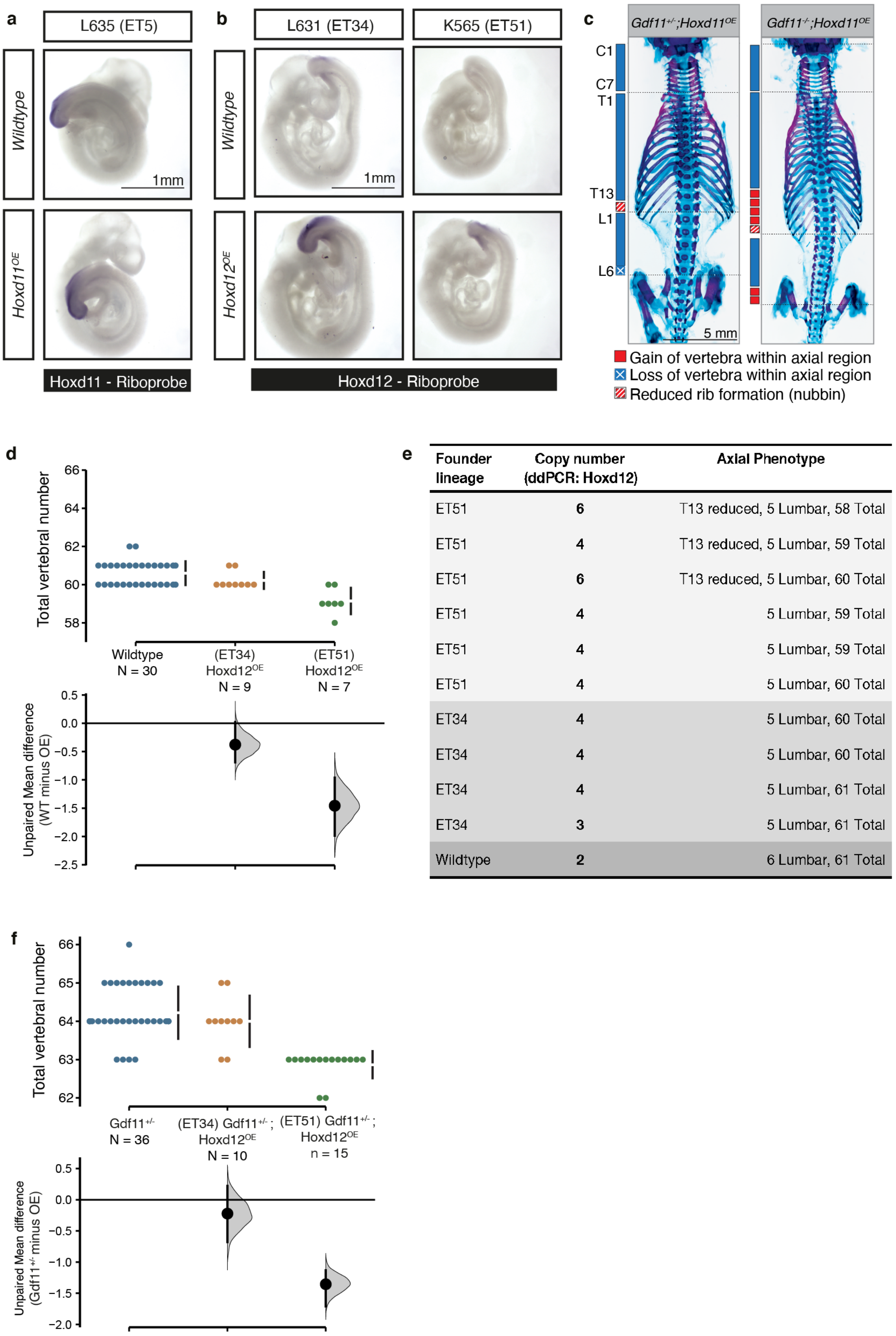

Founder lineage	Copy number (ddPCR: Hoxd12)	Axial Phenotype
ET51	6	T13 reduced, 5 Lumbar, 58 Total
ET51	4	T13 reduced, 5 Lumbar, 59 Total
ET51	6	T13 reduced, 5 Lumbar, 60 Total
ET51	4	5 Lumbar, 59 Total
ET51	4	5 Lumbar, 59 Total
ET51	4	5 Lumbar, 60 Total
ET34	4	5 Lumbar, 60 Total
ET34	4	5 Lumbar, 60 Total
ET34	4	5 Lumbar, 61 Total
ET34	3	5 Lumbar, 61 Total
Wildtype	2	6 Lumbar, 61 Total

Supplementary Figure 4: *In vivo* characterisation of *Hoxd11*^{OE} and *Hoxd12*^{OE} transgenic mouse lines driven by *Cdx2* upstream regulatory elements.

a-b, Characterisation of F1 embryos confirmed germline transmission in one *Hoxd11*^{OE} (OE= overexpressor) **a**, and two *Hoxd12*^{OE} **b**, mouse lines. Whole mount *in situ* hybridisation characterisation of each respective *Hox* gene confirmed transgene expression, with slightly precocious activation and increased levels of *Hox* expression observed in transgene-positive

embryos relative to WT. **c,** Embryonic day (E)18.5 skeletal analysis of *Hoxd11*^{OE} transgenic allele cross-bred with the *Gdf11* mutant line revealed similar changes in the presacral column as seen with the *Hoxd12*^{OE} transgenic allele. C= cervical; T= thoracic; L= lumbar. **d-f,** Quantitative differences in *Hoxd12* copy number correlate with phenotypic variation in axial formulae. **d**, Quantification of total vertebral number within progeny of individual *Hoxd12*^{OE} founder lines. Relative to WT, *Hoxd12*^{OE} founder line ET51 embryos displayed a significant decrease in total vertebral number (TVN). In *Hoxd12*^{OE} founder line ET34 embryos, a trend towards reduced TVN was observed but was not significant. **e**, Correlation of axial formulae and *Hoxd12* copy number for multiple embryos of each founder line indicates that quantitative differences in *Hoxd12* expression levels underlie the phenotypic variation observed. **f**, Quantification of TVN in *Gdf11*$^{+/-}$ embryos, with or without two independent *Hoxd12*^{OE} founder lines (ET34 and ET51). **d, f,** Raw data is presented in the upper plot (vertical error bar = mean and standard deviation). Mean differences relative to shared reference genotype (here WT or *Gdf11*$^{-/-}$) are presented in the lower plot as bootstrap sampling distributions. Each mean difference is depicted as a dot and 95% confidence interval is indicated by the ends of the vertical error bar. n refers to the number of individual animals used for this analysis. Source data are provided as a Source Data file.

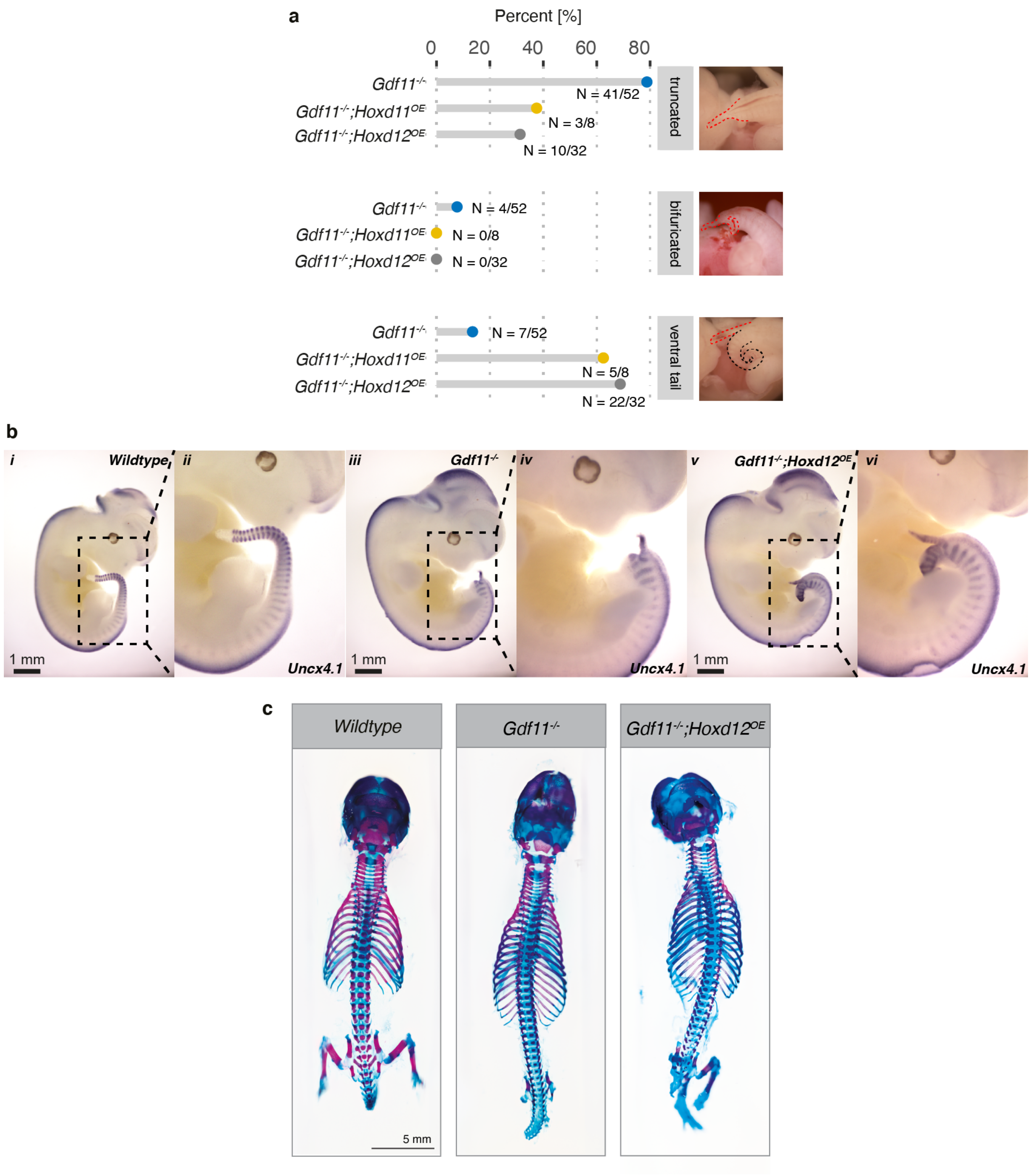

Supplementary Figure 5: Restoration of posterior Hox expression in *Gdf11*$^{-/-}$ embryos supports outgrowth of tail-like structures at mid-gestation.
a, Frequency of observed tail phenotypes across all E12.5 and E13.5 *Gdf11*$^{-/-}$, *Gdf11*$^{-/-}$*;Hoxd11*^{OE} and *Gdf11*$^{-/-}$*;Hoxd12*^{OE} embryos. Source data are provided as a Source Data file. **b**, Whole-mount *in situ* hybridisation of somite marker *Uncx4.1* in E12.5 WT, *Gdf11*$^{-/-}$ and *Gdf11*$^{-/-}$*;Hoxd12*^{OE} embryos, confirming segmented *Uncx4.1*+ tissue forms in the ventral tail of *Gdf11*$^{-/-}$*;Hoxd12*^{OE}. **c**, Embryonic day (E)18.5 skeletal comparison. Caudal truncation in *Gdf11*$^{-/-}$*;Hoxd12*^{OE} embryos was commonly observed immediately after the last sacral element, whereas numerous post-sacral elements commonly formed in *Gdf11*$^{-/-}$ embryos.

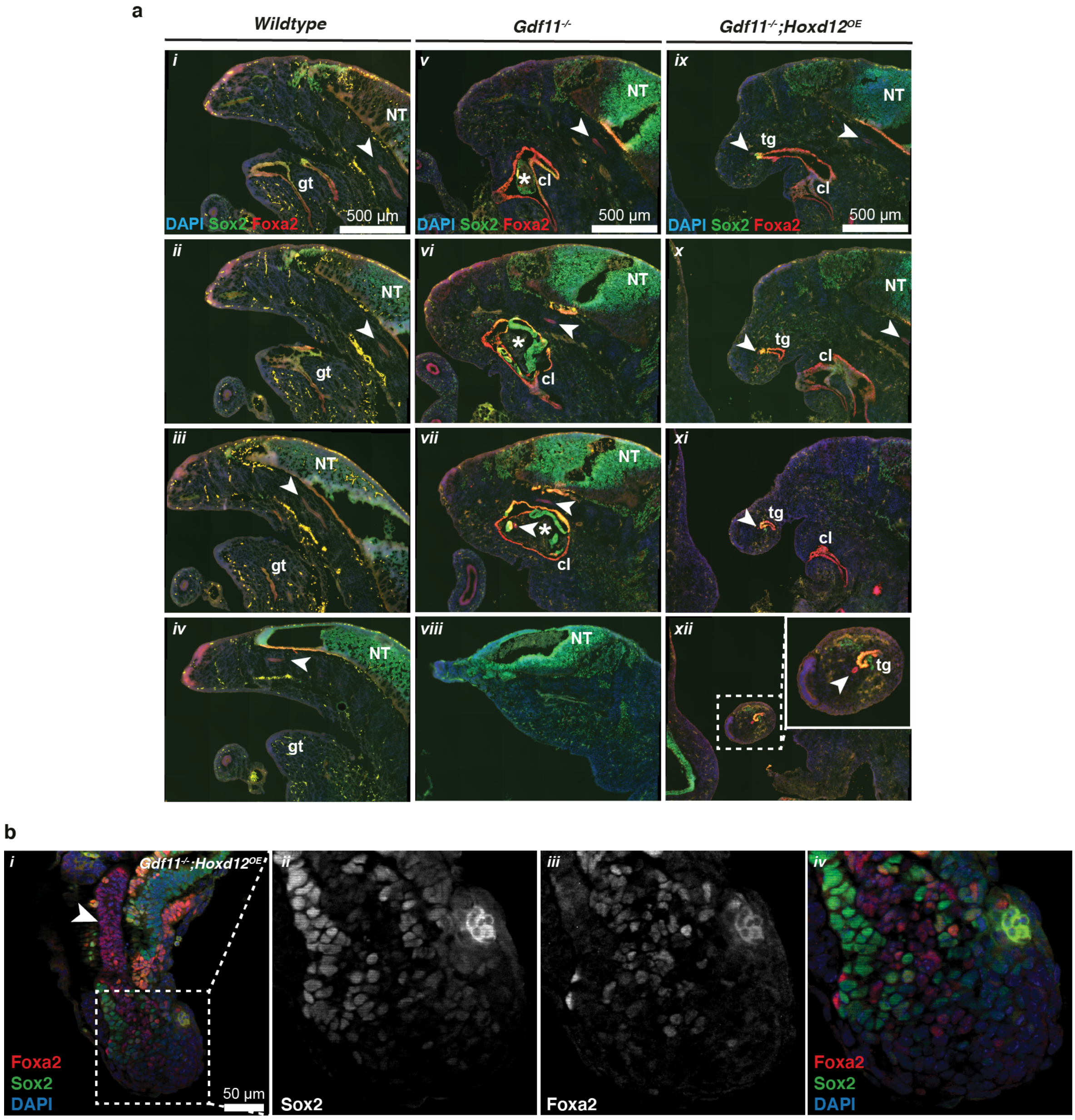

Supplementary Figure 6: Histological analysis reveals molecular, cellular and tissue-level alterations in *Gdf11*$^{-/-}$ and *Gdf11*$^{-/-}$*;Hoxd12*^{OE} embryos.

a, Histological analysis of tail phenotypes in E12.5 WT (n=1) *(i-iv), Gdf11*$^{-/-}$ (n=2) (v-viii) and *Gdf11*$^{-/-}$*;Hoxd12*^{OE} (n=4) (iv-xii) mouse embryos. Serial sagittal sectioning of the embryos presented in whole-mount (Fig. 4a), stained for Foxa2 (red), Sox2 (green) and DAPI (blue). Arrowhead indicates the position of the notochord in all genotypes. Asterisk highlights Sox2+ cells trapped within Foxa2+ cells in *Gdf11*$^{-/-}$ embryos. Sox2+;Foxa2+ co-expression is observed in cells at the distal tip on the tailgut in *Gdf11*$^{-/-}$*;Hoxd12*^{OE} embryos. gt= genital tubercle, cl= cloacal cavity, NT= neural tube, tg= tailgut. n refers to the number of individual animals used for this analysis. **b**, Serial section of ventral tail in Fig. 4c showing the *Gdf11*$^{-/-}$*;Hoxd12*^{OE} tailbud at E12.5. Co-staining for Sox2 (green) and Foxa2 (red) identified single positive cells of either marker and dual positive cells (n=3). All sections are stained for DAPI (blue), arrowhead indicates notochord. n refers to the number of individual animals used for this analysis.

Supplemental Tables

Supplementary Table 1

Skeletal analysis of wild-type, Gdf11-/+ and Gdf11 -/- mice with or without at least five miR-196 alleles deleted.

	Wildtype		TKO*		Gdf11-/+		Gdf11-/+, TKO*		Gdf11-/-		Gdf11-/-, TKO*	
	n	%	n	%	n	%	n	%	n	%	n	%
Presacral vertebrae**												
25	4	*7*	-		-		-		-		-	
26	53	*93*	1	*3*	2	*3*	-		-		-	
27	-		32	*97*	62	*95*	1	*6*	-		-	
28	-		-		1	*2*	6	*35*	-		-	
29	-		-		-		10	*59*	-		-	
33	-		-		-		-		3	*9*	-	
34	-		-		-		-		27	*84*	-	
35	-		-		-		-		2	*6*	-	
37	-		-		-		-		-		1	*25*
39	-		-		-		-		-		3	*75*
Vertebral pattern												
C7 T13 L5	4	*7*	-		-		-		-		-	
C7 T13 L6	53	*93*	1	*3*	1	*2*	-		-		-	
C7 T14 L6	-		11	*33*	60	*92*	1	*6*	-		-	
C7# T14 L7	-		-		1	*2*	-		-		-	
C7 T14 L6***	-		-		1	*2*	-		-		-	
C7 T15 L5	-		21	*64*	1	*2*	-		-		-	
C7 T15 L6***	-		-		1	*2*	-		-		-	
C7 T15 L6	-		-		-		4	*24*	-		-	
C7 T15 L7	-		-		-		1	*6*	-		-	
C7 T16 L5	-		-		-		2	*12*	-		-	
C7 T16 L6	-		-		-		7	*41*	-		-	
C7 T17 L5	-		-		-		2	*12*	-		-	
C7 T18 L8	-		-		-		-		1	*3*	-	
C7 T18 L9	-		-		-		-		22	*69*	-	
C7 T18 L9***	-		-		-		-		2	*6*	-	
C7 T18 L10***	-		-		-		-		3	*9*	-	
C7 T18**** L9	-		-		-		-		1	*3*	-	
C7 T19**** L9	-		-		-		-		1	*3*	-	
C7 T19**** L9***	-		-		-		-		1	*3*	-	
C7 T19**** L10***	-		-		-		-		1	*3*	-	
C7 T21 L9	-		-		-		-		-		1	*25*
C7 T22 L10	-		-		-		-		-		2	*50*
C7 T23 L9	-		-		-		-		-		1	*25*

*Only mice with at least five miR-196 knock-out alleles included. **Vertebrae that had lumbar characteristics on one side and sacral characteristics on the other were scored as sacral. *** Most posterior lumbar element shows lumbar characteristics on one side and sacral characteristics on the other. **** Most posterior thoracic element shows malformed/reduced ribs. # C7 shows thoracic characteristics on one side and cervical characteristics on the other

Supplementary Table 2

Skeletal analysis of wild-type, Gdf11-/+ and Gdf11 -/- mice with and without at least five miR-196 alleles deleted - treated with 0.8mg/kg AGN193109 via oral gavage of pregnant mothers at E7.5/E8.5/E9.5.

	Wildtype + AGN193109		TKO + AGN193109		Gdf11-/+ + AGN193109		Gdf11-/+, TKO + AGN193109		Gdf11-/- + AGN193109		Gdf11-/-, TKO* + AGN193109	
	n	%	n	%	n	%	n	%	n	%	n	%
Presacral vertebrae**												
26	6	67	-		-		-		-		-	
27	3	33	1	17	5	42	-		-		-	
28	-		3	50	7	58	-		-		-	
29	-		2	33	-		1	11	-		-	
30	-		-		-		8	89	-		-	
33	-		-		-		-		3	50	-	
34	-		-		-		-		3	50	-	
37	-		-		-		-		-		1*	50
40	-		-		-		-		-		1	50
Vertebral pattern												
C6 T16*** L5	-		1	17	-		-		-		-	
C6/7 T15*** L6	-		1	17	-		-		-		-	
C7 T13 L6	2	22	-		-		-		-		-	
C7 T13*** L6	4	44	-		-		-		-		-	
C7 T14 L6	2	22	-		2	17	-		-		-	
C7 T14*** L6	1	11	-		2	17	-		-		-	
C7 T15*** L5	-		-		1	8	-		-		-	
C7 T15 L6	-		-		1	8	-		-		-	
C7 T15*** L6	-		1	17	1	8	-		-		-	
C7 T16 L5	-		1	17	-		-		-		-	
C7 T16*** L6	-		1	17	-		1	11	-		-	
C7 T16*** L7	-		-		-		3	33	-		-	
C7 T17*** L6	-		-		-		4	44	-		-	
C7 T18*** L8	-		-		-		-		1	17	-	
C7 T21 L9	-		-		-		-		-		1*	50
C7 T22 L11	-		-		-		-		-		1	50
C7/8 T14 L6	-		-		3	25	-		-		-	
C7/8 T17 L8	-		-		-		-		1	17	-	
C8 T14 L6	-		-		1	8	-		-		-	
C8 T14*** L6	-		-		1	8	-		-		-	
C8 T15*** L6	-		1	17	-		-		-		-	
C8 T16*** L6	-		-		-		1	11	-		-	
C8 T17 L8	-		-		-		-		1	17	-	
C8 T17 L9	-		-		-		-		2	33	-	
C8 T17*** L8	-		-		-		-		1	17	-	

*Mouse with five (out of six) miR-196 alleles knocked out included. **Vertebrae that had lumbar characteristics on one side and sacral characteristics on the other were scored as sacral. *** Most posterior thoracic element shows malformed ribs.

Supplementary Table 3

Skeletal analysis of wild-type, Gdf11-/+ and Gdf11 -/- mice overexpressing either Hoxd11 or Hoxd12 under the Cdx2-Promoter																		
	Gdf11+/+		Gdf11+/+, Cdx2P-Hoxd11		Gdf11+/+, Cdx2P-Hoxd12		Gdf11-/+		Gdf11-/+, Cdx2P-Hoxd11		Gdf11-/+, Cdx2P-Hoxd12		Gdf11-/-		Gdf11-/-, Cdx2P-Hoxd11		Gdf11-/-, Cdx2P-Hoxd12	
	n	%	n	%	n	%	n	%	n	%	n	%	n	%	n	%	n	%
Presacral vertebrae *																		
25	4	*7*	15	*100*	15	*94*	-		-		-		-		-		-	
26	53	*93*	-		1	*6*	2	*3*	23	*96*	21	*81*	-		-		-	
27	-		-		-		62	*95*	1	*4*	5	*19*	-		-		-	
28	-		-		-		1	*2*	-		-		-		-		-	
32	-		-		-		-		-		-		-		-		1	*9*
33	-		-		-		-		-		-		3	*9*	7	*58*	6	*55*
34	-		-		-		-		-		-		27	*84*	5	*42*	4	*36*
35	-		-		-		-		-		-		2	*6*	-		-	
Vertebral pattern																		
C7 T13 L5	4	*7*	6	*40*	11	*69*	-		-		-		-		-		-	
C7 T13** L5	-		9	*60*	3	*19*	-		-		-		-		-		-	
C7 T13** L6***	-		-		1	*6*	-		-		-		-		-		-	
C7 T13 L6	53	*93*	-		1	*6*	1	*2*	-		-		-		-		-	
C7 T14 L6	-		-		-		60	*92*	1	*4*	5	*19*	-		-		-	
C7# T14 L7	-		-		-		1	*2*	-		-		-		-		-	
C7 T14 L6***	-		-		-		1	*2*	-		4	*15*	-		-		-	
C7 T15 L5	-						1	*2*					-					
C7 T15 L6***	-						1	*2*					-					
C7 T14 L5	-		-		-		-		12	*50*	4	*15*	-		-		-	
C7 T14** L5	-		-		-		-		11	*46*	11	*42*	-		-		-	
C7 T14** L6***	-		-		-		-		-		2	*8*	-		-		-	
C7 T17 L9	-		-		-		-		-		-		-		1	*8*	-	
C7 T18 L8	-		-		-		-		-		-		1	*3*	2	*17*	-	
C7 T18 L9	-		-		-		-		-		-		22	*69*	5	*42*	2	*18*
C7 T18 L9***	-		-		-		-		-		-		2	*6*	1	*8*	1	*9*
C7 T18 L10***	-		-		-		-		-		-		3	*9*	-		2	*18*
C7 T18** L9	-		-		-		-		-		-		1	*3*	-		-	
C7 T18 L8	-		-		-		-		-		-		-		-		1	*9*
C7 T18** L8	-		-		-		-		-		-		-		-		3	*27*
C7 T18** L8***	-		-		-		-		-		-		-		-		1	*9*
C7 T18** L9***	-		-		-		-		-		-		-		3	*25*	1	*9*
C7 T19** L9	-		-		-		-		-		-		1	*3*	-		-	
C7 T19** L9***	-		-		-		-		-		-		1	*3*	-		-	
C7 T19** L10***	-		-		-		-		-		-		1	*3*	-		-	

* Vertebrae that had lumbar characteristics on one side and sacral characteristics on the other were scored as sacral. ** Most posterior thoracic element shows malformed/reduced ribs. *** Most posterior lumbar element shows lumbar characteristics on one side and sacral characteristics on the other. # C7 shows thoracic characteristics on one side and cervical characteristics on the other

Supplementary Table 4

Unpaired mean differences in total vertebral number counts

Genotypes compared	Unpaired mean difference	[Confidence interval width: lower bound; upper bound]
WT and TKO	1.1	[95.0%CI: 0.72, 1.43]
WT and Gdf11$^{+/-}$	3.56	[95.0%CI: 3.28, 3.82]
WT and TKO; Gdf11$^{+/-}$	5.13	[95.0%CI: 4.52, 5.58]
WT and WT +AGN	1.44	[95.0%CI 1.22, 1.62]
TKO and TKO +AGN	2.01	[95.0%CI 1.35, 3.41]
Gdf11$^{+/-}$ and Gdf11$^{+/-}$ +AGN	1.24	[95.0%CI 0.789, 1.77]
TKO; Gdf11$^{+/-}$ and TKO; Gdf11$^{+/-}$ +AGN	1.81	[95.0%CI 1.17, 2.75]
WT and Hoxd11^{OE}	-0.417	[95.0%CI -0.751, -0.0714]
WT and Hoxd12^{OE}	-0.81	[95.0%CI -1.28, -0.432]
Gdf11$^{+/-}$ and Gdf11$^{+/-}$;Hoxd11^{OE}	-0.476	[95.0%CI -0.875, -0.195]
Gdf11$^{+/-}$ and Gdf11+/-;Hoxd12^{OE}	-0.803	[95.0%CI -1.13, -0.451]
WT and Hoxd12^{OE} (ET34)	-0.378	[95.0%CI -0.689, 0.0222]
WT and Hoxd12^{OE} (ET51)	-1.46	[95.0%CI -1.99, -0.962]
Gdf11$^{+/-}$ and Gdf11$^{+/-}$;Hoxd12^{OE} (ET34)	-0.222	[95.0%CI -0.678, 0.222]
Gdf11$^{+/-}$ and Gdf11$^{+/-}$;Hoxd12^{OE} (ET51)	-1.36	[95.0%CI -1.71, -1.13]

Supplementary Table 5

Taqman Probes used for Biomark Fluidigm

Gene target	Probe ID	Gene target	Probe ID
Gapdh	Mm03302249_g1	*Hoxb6*	Mm00433970_m1
Gapdh	Mm99999915_g1	*Hoxb7*	Mm00650702_m1
B-actin	Mm00446971_m1	*Hoxb8*	Mm00439368_m1
Ubc	Mm01205647_g1	*Hoxb9*	Mm01700220_m1
Tbp	Mm02525934_g1	*Hoxc10*	Mm01305933_m1
Hoxa1	Mm00439359_m1	*Hoxc11*	Mm01305934_m1
Hoxa10	Mm00433966_m1	*Hoxc12*	Mm00807029_m1
Hoxa11	Mm00439360_m1	*Hoxc13*	Mm00802798_m1
Hoxa13	Mm00433967_m1	*Hoxc4*	Mm00442838_m1
Hoxa2	Mm00439361_m1	*Hoxc5*	Mm00433971_m1
Hoxa3	Mm01326402_m1	*Hoxc6*	Mm01307713_m1
Hoxa4	Mm01335255_g1	*Hoxc8*	Mm00439369_m1
Hoxa5	Mm04213381_s1	*Hoxc9*	Mm00433972_m1
Hoxa6	Mm00550244_m1	*Hoxd1*	Mm00439370_g1
Hoxa7	Mm00657963_m1	*Hoxd10*	Mm00442839_m1
Hoxa9	Mm00439364_m1	*Hoxd11*	Mm02602515_mH
Hoxb1	Mm00515118_g1	*Hoxd12*	Mm01962622_s1
Hoxb13	Mm00433968_m1	*Hoxd13*	Mm00433973_m1
Hoxb2	Mm04209931_m1	*Hoxd3*	Mm00439371_m1
Hoxb3	Mm04182289_s1	*Hoxd4*	Mm01333847_g1
Hoxb4	Mm00657964_m1	*Hoxd8*	Mm03016337_m1
Hoxb5	Mm00657672_m1	*Hoxd9*	Mm00442840_m1

Supplementary Table 6

Primer sequences used in this study

Primer	Direction	Used for	Sequence 5'-3'	Source
Hoxb1	F	qPCR	GGCAGGAGTTGGGAAATGTA	Kondrashov et al. 2011
Hoxb1	R	qPCR	GGCTGACTCCAGATCAAAGC	Kondrashov et al. 2011
Hoxb8	F	qPCR	TGCGCCCCAATTATTATGAC	Kondrashov et al. 2011
Hoxb8	R	qPCR	TTCTGCTGGTAGGGAGCTGT	Kondrashov et al. 2011
Hoxc8	F	qPCR	TTTGGTTCCAGAATCGAAGG	Kondrashov et al. 2011
Hoxc8	R	qPCR	GGGGGCTGATTTTCTCTCTC	Kondrashov et al. 2011
Hoxd13	F	qPCR	CGACATGGTGTCCACTTTTG	Kondrashov et al. 2011
Hoxd13	R	qPCR	TGGTGTAAGGCACCCTTTTC	Kondrashov et al. 2011
Hoxc9	F	qPCR	ACGTGGACTCGCCTCATCTCT	Kondrashov et al. 2011
Hoxc9	R	qPCR	GCCGTAAGGGTGATAGACCA	Kondrashov et al. 2011
Hoxd12	F	qPCR	GCATGAAACAGGAGCCTAGC	Kondrashov et al. 2011
Hoxd12	R	qPCR	CCTTTCCTTCCTGCAGAGTG	Kondrashov et al. 2011
Sox1	F	qPCR	CACCGCTACGACATGGGC	In-house
Sox1	R	qPCR	TAAGGGATGCCGCCGTAG	In-house
Sox2	F	qPCR	GCACATGAACGGCTGGAGCAACG	In-house
Sox2	R	qPCR	TGCTGCGAGTAGGACATGCTGTAG	In-house
Tbra	F	qPCR	GGCTGTTGGGTAGGGAGTCA	In-house
Tbra	R	qPCR	GGAACATCCTCCTGCCGTTCTT	In-house
Hoxc12	F	ISH Probe	GAAATGGGCGAGCATAATCTCC	In-house
Hoxc12	R	ISH Probe	CTCCACCAAGGCAAGCTTAGTC	In-house
Hoxd12	F	ddPCR	ACTTTTCCAACCTGAGAGCC	In-house
Hoxd12	R	ddPCR	CGGTCAAGTAAGGCTGAGAG	In-house
Hoxd12	Probe	ddPCR	/56-FAM/ TGCACCCCT /ZEN/ GCGCAGCCTGC /3IABkFQ/	In-house
Rpp30	F	ddPCR	CTTTGAACTTGTCTATGGTCCT	In-house
Rpp30	R	ddPCR	GCATCAAATTGAGGGCATTG	In-house
Rpp30	Probe	ddPCR	/5-HEX/ TGTGTACCT /ZEN/ TCTCATCGTTGCATC /3IABkFQ/	In-house
