# Supplementary material for: Breaking constraint of mammalian axial formulae
Source: Nat Commun. 2022 Jan 11;13:243. doi: 10.1038/s41467-021-27335-z (PMC8752674; doi:10.1038/s41467-021-27335-z)
Supplement: Supplementary file 3 — Description of Additional Supplementary Files [file 41467_2021_27335_MOESM3_ESM.pdf]

File Name: Supplementary Movie 1
Description: Wildtype E13.5 mouse embryo, caudal region, stained for Sox2 (red), T/Brachyury (T;Bra; green) and Foxa2 (blue) proteins. 3-Disco clearing, Ultramicroscope II imaging.

File Name: Supplementary Movie 2
Description: *Gdf11*$^{-/-}$;*Hoxd12*^{OE}*_1* embryo, caudal region, stained for Sox2 (red), T/Brachyury (T;Bra; green) and Foxa2 (blue) proteins. 3-Disco clearing, Ultramicroscope II imaging.

File Name: Supplementary Movie 3
Description: *Gdf11*$^{-/-}$;*Hoxd12*^{OE}*_2* embryo, caudal region, stained for Sox2 (red), T/Brachyury (T;Bra; green) and Foxa2 (blue) proteins. 3-Disco clearing, Ultramicroscope II imaging.
