# Supplementary material for: Breaking constraint of mammalian axial formulae
Source: Nat Commun. 2022 Jan 11;13:243. doi: 10.1038/s41467-021-27335-z (PMC8752674; doi:10.1038/s41467-021-27335-z)
Supplement: Supplementary file 7 — Reporting Summary [file 41467_2021_27335_MOESM7_ESM.pdf]

nature portfolio

Corresponding author(s): Edwina McGlinn

Last updated by author(s): 29/10/21

Reporting Summary

Nature Portfolio wishes to improve the reproducibility of the work that we publish. This form provides structure for consistency and transparency in reporting. For further information on Nature Portfolio policies, see our Editorial Policies and the Editorial Policy Checklist.

Statistics

For all statistical analyses, confirm that the following items are present in the figure legend, table legend, main text, or Methods section.

n/a	Confirmed	
☐	☒	The exact sample size (*n*) for each experimental group/condition, given as a discrete number and unit of measurement
☐	☒	A statement on whether measurements were taken from distinct samples or whether the same sample was measured repeatedly
☒	☐	The statistical test(s) used AND whether they are one- or two-sided<br>*Only common tests should be described solely by name; describe more complex techniques in the Methods section.*
☒	☐	A description of all covariates tested
☒	☐	A description of any assumptions or corrections, such as tests of normality and adjustment for multiple comparisons
☐	☒	A full description of the statistical parameters including central tendency (e.g. means) or other basic estimates (e.g. regression coefficient) AND variation (e.g. standard deviation) or associated estimates of uncertainty (e.g. confidence intervals)
☐	☒	For null hypothesis testing, the test statistic (e.g. *F*, *t*, *r*) with confidence intervals, effect sizes, degrees of freedom and *P* value noted<br>*Give P values as exact values whenever suitable.*
☒	☐	For Bayesian analysis, information on the choice of priors and Markov chain Monte Carlo settings
☒	☐	For hierarchical and complex designs, identification of the appropriate level for tests and full reporting of outcomes
☒	☐	Estimates of effect sizes (e.g. Cohen's *d*, Pearson's *r*), indicating how they were calculated

Our web collection on statistics for biologists contains articles on many of the points above.

Software and code

Policy information about availability of computer code

Data collection	Lightcycler 480 (Roche) software, QX200 droplet reader (Bio-Rad, USA) software, MIRAX Scan software, AxioVisionRel.4.8 image acquisition software, MosaiX, ZereneStacker, Ultramicroscope 2 software(LaVision, Miltenyi Biotec GMbH), Fluidigm Biomark software
Data analysis	ImageJ2.0, DABEST R-package, QuantaSoft Analysis Pro Version 1.0 (Bio-Rad, USA), qPCR-Biomark script (https://github.com/jpouch/qPCR-Biomark), Imaris (v9.6; BitPlane, Oxford Instrument), Adobe Illustrator (v25.2.0), Adobe Photoshop (v22.2.0), ggplot2(v3.3.2), MIRAMAX Viewer Software (v1.12)

For manuscripts utilizing custom algorithms or software that are central to the research but not yet described in published literature, software must be made available to editors and reviewers. We strongly encourage code deposition in a community repository (e.g. GitHub). See the Nature Portfolio guidelines for submitting code & software for further information.

Data

Policy information about availability of data

All manuscripts must include a data availability statement. This statement should provide the following information, where applicable:

- Accession codes, unique identifiers, or web links for publicly available datasets
- A description of any restrictions on data availability
- For clinical datasets or third party data, please ensure that the statement adheres to our policy

Summary tables of skeletal scoring data are available as supplementary tables 1-3, numerical source data corresponding to the Fluidigm qPCR data is available as supplementary data in the Source Data excel file. Supplementary Movies are available under the link: https://figshare.com/s/78383c7beb9b85c959bf

March 2021

Field-specific reporting

Please select the one below that is the best fit for your research. If you are not sure, read the appropriate sections before making your selection.

[x] Life sciences [ ] Behavioural & social sciences [ ] Ecological, evolutionary & environmental sciences

For a reference copy of the document with all sections, see nature.com/documents/nr-reporting-summary-flat.pdf

Life sciences study design

All studies must disclose on these points even when the disclosure is negative.

Sample size

No statistical method was used to predetermine sample size.
For qualitative determination of gene expression differences using whole-mount in situ hybridisation, a minimum of 3 biologically distinct samples per time-point and per genotype were assessed.
For quantitative determination of gene expression using Fluidigm, a minimum of 3 biological samples per genotype and per time-point were assessed.
For quantitative determination vertebral number and morphology, a minimum of 6 and maximum of 65 biological distinct samples were used per genotype, with the exception of Gdf11-/-;miR-196-TKO+AGN where n= 2.

Data exclusions

For gene expression analyses, Ct values greater than the manufacturer recommended threshold were excluded.
The only sample exclusion for skeletal analysis was if the skeleton was damaged during the process of collection to the point where a complete dataset could not be collected for the specimen.

Replication

Each embryo/skeleton is considered an individual sample, and thus replicate number = sample size. All attempts at replication were successful.
For whole-mount in situ hybridisation, a minimum of 3 biologically distinct replicates per time-point and per genotype were assessed.
For Fluidigm qPCR, a minimum of 3 independent biological replicates per genotype and per time-point were assessed.
For skeletal phenotyping, a minimum of 6 and maximum of 65 biological distinct replicates were used per genotype, with the exception of Gdf11-/-;miR-196-TKO+AGN where n= 2.

Randomization

Allocation of experimental groups was set out by mouse genotypes.

Blinding

All skeletons were scored for vertebral number and morphology by 2 researchers, blinded to genotype. Any instance where there was discrepancy in result, the skeleton in question was scored by a third individual, also blinded to genotype. For experiments other than those mentioned here, the investigators were blinded to group allocation during data collection and/or analysis.

Reporting for specific materials, systems and methods

We require information from authors about some types of materials, experimental systems and methods used in many studies. Here, indicate whether each material, system or method listed is relevant to your study. If you are not sure if a list item applies to your research, read the appropriate section before selecting a response.

Materials & experimental systems

n/a	Involved in the study	
[ ]	[x]	Antibodies
[ ]	[x]	Eukaryotic cell lines
[x]	[ ]	Palaeontology and archaeology
[ ]	[x]	Animals and other organisms
[x]	[ ]	Human research participants
[x]	[ ]	Clinical data
[x]	[ ]	Dual use research of concern

Methods

n/a	Involved in the study	
[x]	[ ]	ChIP-seq
[x]	[ ]	Flow cytometry
[x]	[ ]	MRI-based neuroimaging

Antibodies

Antibodies used

Primary antibodies: rabbit anti-T/Brachyury (T) antibody (AbCam, Ab209665), rat anti-SOX2 antibody (AbCam, Ab92494), rat anti-Sox2 (1:250, FisherScientific, Btjce), rabbit anti-Foxa2 (SevenHills Bioreagents, WRAB-1200), goat anti-T/Brachyury (1:100, R&D, AF2085). Secondary antibodies: anti-rat AlexaFluor 488 (ThermoFisher, A212208), anti-rabbit AlexaFluor 555 (ThermoFisher, A31572), donkey anti-goat-AlexaFluor-647 (1:1000, ThermoFisher, A-21447), anti-rat-AlexaFluor-555 (1:1000, ThermoFisher, A-21434), goat anti-rabbit-AlexaFluor-790 (1:1000, Invitrogen, A11369).

Validation

All primary antibodies used in this study were commercially purchased and have been used in previous studies investigating similar or the same cell types under analysis here (see [AbCam, Ab209665] - Moris et al. 2020, https://doi.org/10.1038/s41586-020-2383-9; , [AbCam, Ab92494] [R&D, AF2085] - Aires et al. 2019, https://doi.org/10.1016/j.devcel.2018.12.004; [FisherScientific, Btjce] - Chen et al. 2017, https://doi.org/10.1002/cne.24206; [evenHills Bioreagents, WRAB-1200] - Johansson et al. 2015, https://doi.org/10.1242/

dev.126581). Additional technical information is available on the manufactures websites. As antibody lots can be variable, each antibody was verified in-house to replicate the correct expression pattern and cell location within the tissues tested, and a secondary antibody only control is routinely incorporated into each experiment to test for non-specific binding.

Eukaryotic cell lines

Policy information about cell lines

Cell line source(s)	Bruce4 mouse embryonic stem cell line isolated from C57BL/6 mouse strain (Abbondanzo et al., 1993).<br>Two in-house generated iPSC lines were derived from the miR-196 triple knockout mouse strain (Wong et al., PNAS, 2015) or an isogenic wildtype mouse line.
Authentication	iPSC cell lines were authenticated for pluripotency by teratoma assay, see Supplementary Figure 1 of this study.
Mycoplasma contamination	All ESC, iPSC and mouse embryonic fibroblast for feeder layers were routinely tested and confirmed mycoplasma free.
Commonly misidentified lines<br>(See ICLAC register)	Only wildtype ESCs and in-house generated iPSCs were used.

Animals and other organisms

Policy information about studies involving animals; ARRIVE guidelines recommended for reporting animal research

Laboratory animals	Species: Mus musculus<br>Common strains used in this study: Wildtype (C57BL6), Gdf11-KO, miR196-TKO, Cdx2P-Hoxd11, Cdx2P-Hoxd12. Adult and embryonic stages were used and described in the text for each figure.<br>NOD/Scid mice were used for teratoma assay. Here, mice of either sex, and of 8 week or older were used.<br><br>Housing: All mice were housed as outlined in the - Code of Practice for the Housing and Care of Laboratory Mice, Rats, Guinea Pigs and Rabbits (Agriculture Victoria) - 12 hour day-night cycle, temperature (degrees Celsius) 18-24, relative humidity (%) 40-70 and maximum light (lux) 350.<br><br>Animal sex has not previously been shown to impact any experimental output assessed in this study and thus was not assessed.
Wild animals	No wild animals were used in this study.
Field-collected samples	No field-collected samples were used in this study.
Ethics oversight	All animal procedures were performed in accordance with the Australian Code of Practice for the Care and Use of Animals for Scientific Purposes (2013). Experiments were approved by the Monash Animal Ethics Committee under project numbers MARP/2011/012, MARP/2015/168 and MARP/2015/123.

Note that full information on the approval of the study protocol must also be provided in the manuscript.
